# Personal

**Published:** 1903-11

**Authors:** 


### PERSONAL.

DR. WILLIAM H. THORNTON, of Buffalo, was elected president of the New York State Medical Association, at its annual meeting, held at New York, October 19-22, 1903.

DR. CHAUNCEY PELTON SMITH, of Buffalo, has been appointed medical officer of the Lackawanna Steel Company at West Seneca.

DR. WALTER D. GREENE, health commissioner, and Dr. William G. Bissell, bacteriologist of the department of health, attended the annual meeting of the American Public Health Association, at Washington, D. C., October 26-30, 1903, as the official delegates from the Buffalo department of health.

DR. R. C. TABER, of Tonawanda, will soon remove to Jackson, Mich., where he will continue in the future the practice of his profession. With this object in view he has resigned the office of health physician at Tonawanda.

DR. LAWRENCE HENDEE, graduate of the University of Buffalo, 1897, and for several years past an instructor in anatomy at Cornell University, has been appointed volunteer assistant in surgery at the University of Koenigsberg, Germany. Dr. Hendee has been in Berlin for several months pursuing surgical studies.

DR. CARLOS F. MACDONALD, of New York, has removed his office from 85 Madison avenue to 29 East 44th street. Hours, from 10 a.m. until 12 m., Sundays excepted, and by appointment. Telephone 4588-38th St.

